# A feasibility of computational drug screening for Fuchs endothelial corneal dystrophy

**DOI:** 10.1038/s41598-025-95003-z

**Published:** 2025-04-26

**Authors:** Itsuki Oka, Yoshiaki Toyokawa, Kouta Imai, Tatsuya Nakagawa, Theofilos Tourtas, Ursula Schlötzer-Schrehardt, Friedrich Kruse, Noriko Koizumi, Naoki Okumura

**Affiliations:** 1 Department of Biomedical Engineering, Faculty of Life and Medical Sciences, Doshisha University, Kyotanabe, 610-0394 Japan; 2 Department of Ophthalmology, University of Erlangen-Nürnberg, Erlangen, Germany

**Keywords:** Corneal diseases, Hereditary eye disease

## Abstract

Fuchs endothelial corneal dystrophy (FECD) remains a leading cause of corneal blindness globally, with corneal transplantation being the primary treatment. FECD is characterized by the formation of guttae, extracellular matrix (ECM) deposits beneath the corneal endothelium, and progressive endothelial cell loss. These pathological changes cause visual deterioration through light scattering by guttae and corneal edema due to endothelial cell loss. However, limitations such as donor shortage and graft failure necessitate alternative therapeutic approaches. We employed computational drug screening using three platforms (L1000FWD, L1000CDS^2^, and SigCom LINCS) to identify compounds capable of normalizing FECD-associated differentially expressed genes (DEGs). Analysis of transcriptome data from FECD patients with *TCF4*trinucleotide repeat expansion identified 706 upregulated and 962 downregulated genes. The screening platforms identified 200, 35, and 76 compounds through L1000FWD, L1000CDS^2^, and SigCom LINCS, respectively, with five compounds commonly predicted across all platforms. Among these, LDN193189 and cercosporin were selected for further evaluation based on availability and lack of cytotoxicity. Both compounds significantly decreased the expression of ECM-related genes (*FN1*, *MATN3*, *BGN*, and *LTBP2*) in FECD cell models and suppressed TGF-β-induced fibronectin expression. Additionally, both compounds reduced aggresome formation to normal control levels, suggesting protection against endoplasmic reticulum stress-induced cell death. This study demonstrates the feasibility of computational drug screening for identifying therapeutic candidates for FECD, with LDN193189 and cercosporin showing promise in normalizing FECD-associated pathological changes.

**Supplementary Information:**

The online version contains supplementary material available at 10.1038/s41598-025-95003-z.

## Introduction

Fuchs endothelial corneal dystrophy (FECD), a progressive, bilateral disorder that affects the corneal endothelium, leads to corneal edema and visual impairment. The pathogenesis of FECD involves the formation of guttae and the progressive loss of endothelial cells. These pathological changes result in visual deterioration through two mechanisms: the guttae cause visual impairment through light scattering, while endothelial cell loss leads to corneal edema and more severe vision loss^1–3^. Corneal transplantation is the primary treatment; however, it has several limitations, including a shortage of donor corneas, risk of rejection, and potential graft failure due to ongoing endothelial cell damage. Therefore, alternative therapeutic approaches are urgently needed^2,4–7^.

One feature of FECD is a unique gene expression signature in the corneal endothelium, as revealed by comparative transcriptome analyses between FECD patients and healthy controls across several studies^8–10^. Our previous transcriptomic analysis identified 2,366 differentially expressed genes (DEGs) in the FECD-affected corneal endothelium compared to control tissue, with 1,092 genes showing increased expression and 1,274 showing decreased expression^9^. Functional analysis of these DEGs using gene ontology revealed significant enrichment in pathways related to extracellular matrix (ECM) organization, oxidative stress response, and apoptotic signaling^9^.

Notably, these enriched pathways align with the known pathological features of FECD, as ECM dysregulation contributes to guttae formation, while oxidative stress and enhanced apoptotic signaling are established drivers of endothelial cell loss^1–3^. This concordance between our transcriptomic findings and recognized disease mechanisms validates the pathological relevance of the observed gene expression alterations in FECD. In the present study, we employed three computational drug screening platforms (L1000FWD, L1000CDS^2^, and SigCom LINCS)^11–13^ to identify compounds capable of normalizing these DEGs in FECD corneal endothelium—specifically, compounds that could downregulate overexpressed genes and upregulate underexpressed genes. We also used in vitro studies to evaluate the therapeutic potential of drug candidates identified in common across all three in silico platforms, focusing particularly on the ability of the drugs to modulate pathological ECM expression in FECD. This approach allowed us to validate the utility of in silico drug screening methodologies.

## Results

### Computational drug screening using L1000FWD, L1000CDS^2^, and SigCom LINCS

**Fig. 1:**
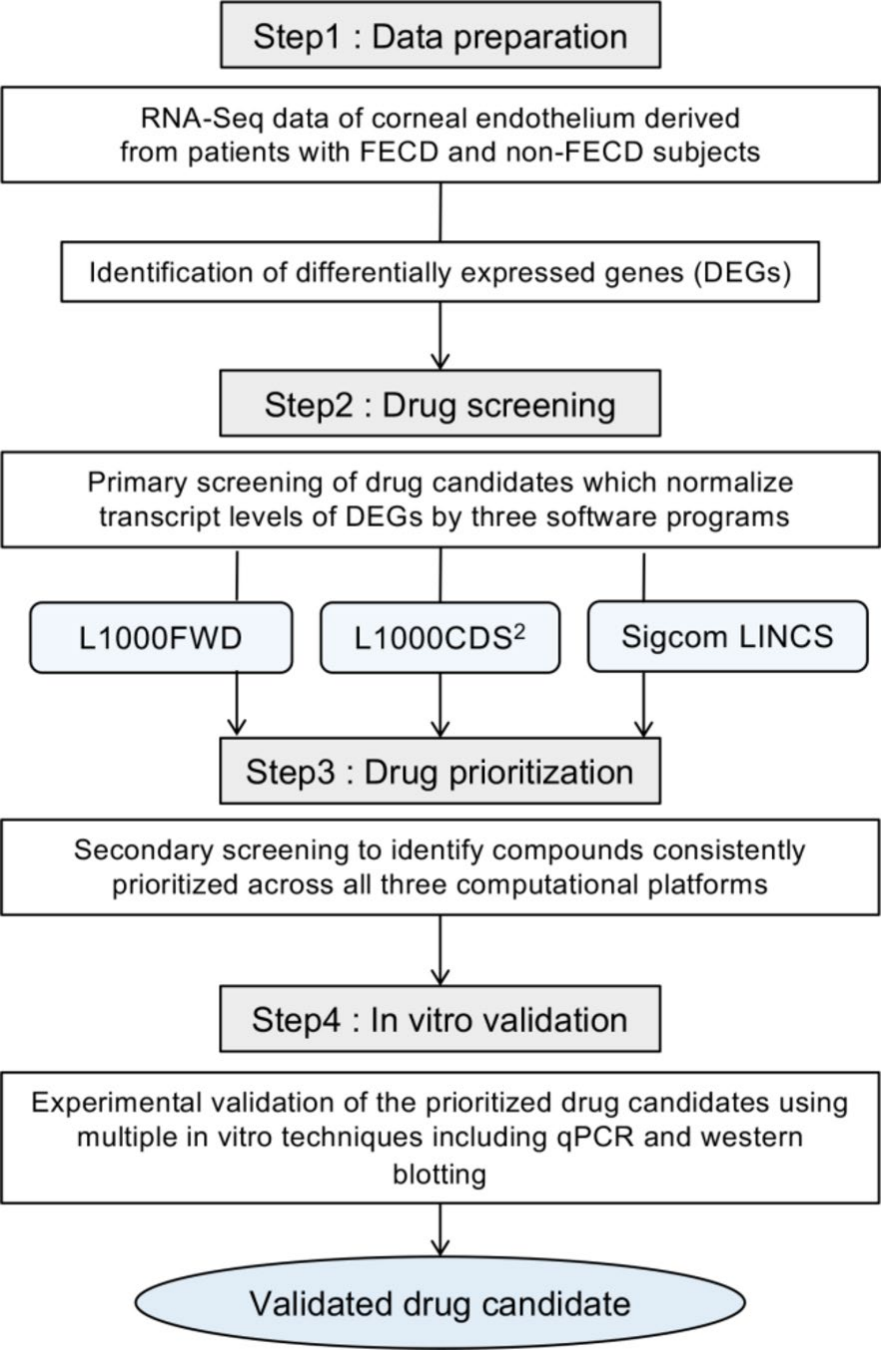
Schematic workflow of drug candidate identification for FECD treatment. Flowchart illustrating the systematic four-step approach taken to identify therapeutic candidates for FECD. Step 1 (Data preparation) began with RNA-Seq analysis of corneal endothelium from FECD patients and non-FECD subjects, followed by identification of differentially expressed genes (DEGs). Step 2 (Drug screening) involved primary screening using three computational platforms (L1000FWD, L1000CDS², and SigCom LINCS) to identify drug candidates that could normalize DEG transcript levels. Step 3 (Drug prioritization) consisted of secondary screening to identify compounds consistently prioritized across all three computational platforms. Step 4 (In vitro validation) evaluated the therapeutic potential of the prioritized drug candidates through experimental validation using multiple in vitro techniques including qPCR and western blotting, ultimately leading to validated drug candidates for FECD treatment.

**Fig. 2:**
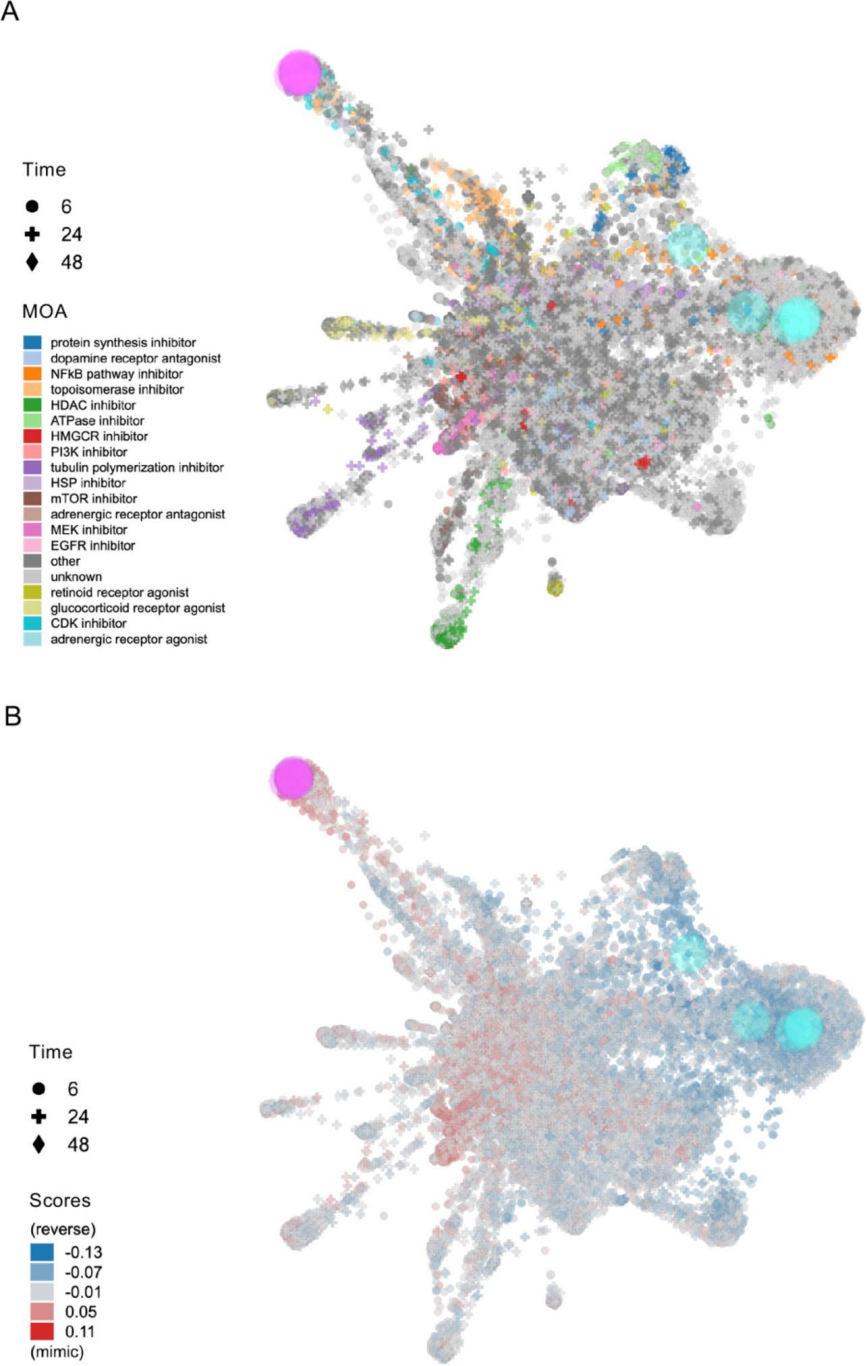
L1000FWD-based visualization of drug candidates for FECD gene expression reversal. (**A**) Drug landscape visualization generated by L1000FWD analysis of differentially expressed genes (DEGs) from FECD patients with TCF4 trinucleotide repeat expansion (TNR > 50) compared to non-FECD subjects (706 upregulated and 962 downregulated genes). The plot displays 16,849 compounds clustered by their mode of action (MOA), represented by the different colors indicated in the legend. The shape of each plot represents the duration of drug exposure (Time) in the corresponding cell line (6, 24, or 48 h) as shown in the legend. (**B**) Score-based visualization of 10,655 identified drug candidates. The color gradient represents the compound effects on FECD-associated gene expression, where blue indicates reversers (compounds that normalize dysregulated gene expression toward the expression observed in non-FECD patterns) and red indicates mimickers (compounds that enhance FECD-associated expression patterns). The color intensity corresponds to the magnitude of effect ( −0.13 to 0.11). These visualizations were generated by the authors using the L1000FWD web-based tool ( https://maayanlab.cloud/L1000FWD/ ), which is freely available for academic research under open access principles. The visualizations shown are direct outputs from our analysis using this tool and are displayed under academic fair use principles and in accordance with the tool’s terms of use.

We conducted primary screening using all three platforms (L1000FWD, L1000CDS^2^, and SigCom LINCS), followed by secondary screening to identify compounds common across these platforms, as part of our overall research workflow shown in Fig. 1. The L1000FWD platform, containing data on 16,849 compounds, visualized potential therapeutic candidates based on their mechanisms of action. Analysis of 1,668 DEGs from FECD cases with TNR expansion identified 10,655 potential drug candidates. The visualization map categorized compounds by their mode of action, including protein synthesis inhibitors, dopamine receptor antagonists, NFKB pathway inhibitors, and various other drug classes (Fig. 2A). The platform’s scoring system (−0.13 to 0.11) distinguished between reverser compounds (blue, negative scores) that normalize dysregulated gene expression and mimicker compounds (red, positive scores) that potentially exacerbate the disease signature (Fig. 2B). The top 200 reverser compounds were selected for subsequent analysis.

**Fig. 3:**
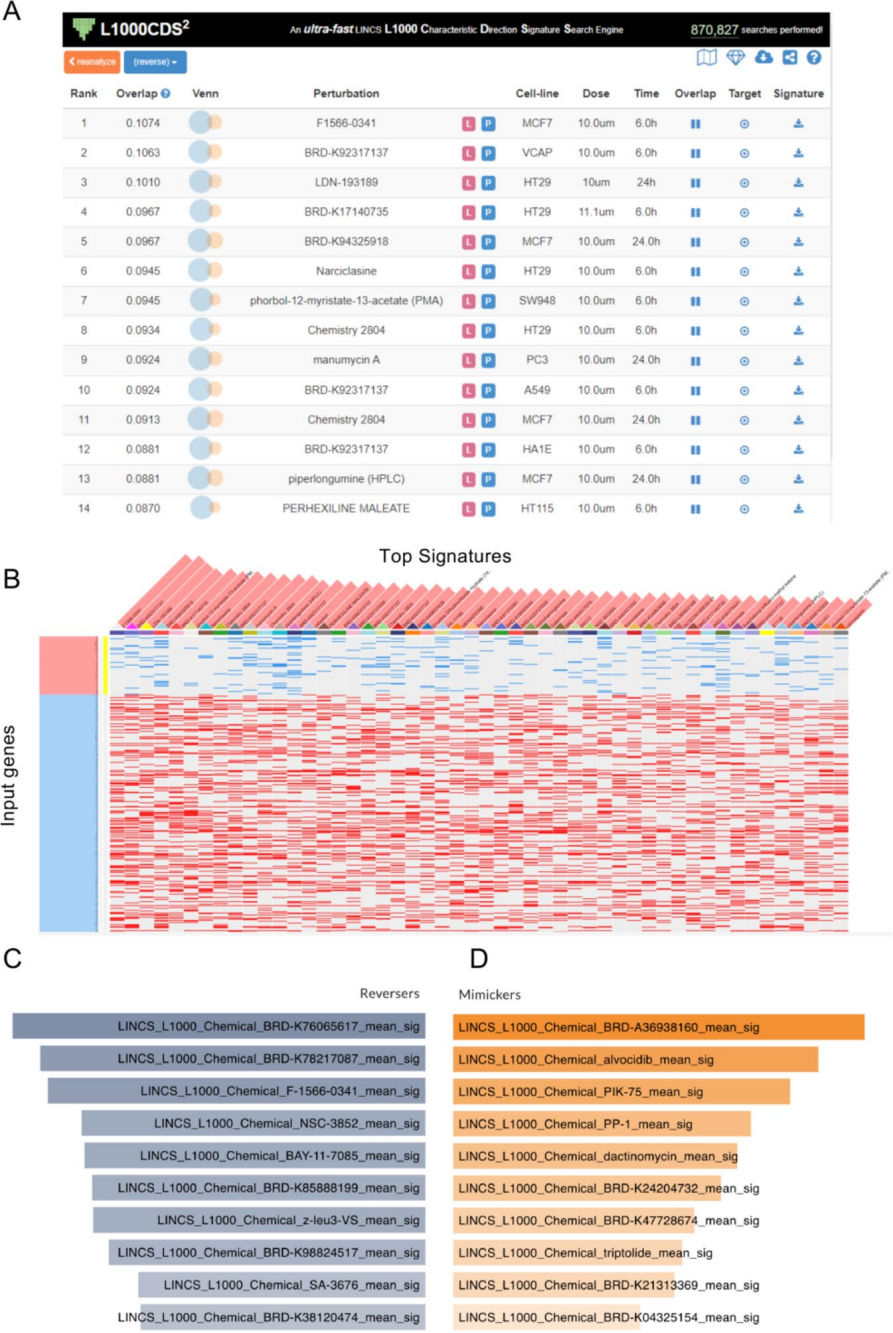
Computational drug screening results from L1000CDS^2 ^and SigCom LINCS platforms. (**A**) L1000CDS^2 ^analysis results showing the top 14 drug candidates identified from screening of FECD-associated DEGs (706 upregulated and 962 downregulated genes from FECD patients with TCF4 TNR > 50). The table displays comprehensive information, including rank, search score, gene overlap visualization, perturbation details, and experimental conditions (cell line, dose, and duration of treatment). (**B**) Heat map visualization of gene expression signatures for the top 50 drug candidates identified by L1000CDS^2^. The map illustrates the impact of each compound on FECD-associated DEGs, with rows representing input genes and columns representing drug candidates. Color intensity indicates the degree of expression changes (red: upregulation; blue: downregulation). (C, D) SigCom LINCS analysis results presenting the top 10 compounds categorized as (**C**) reversers (compounds that normalize FECD gene expression patterns) and (**D**) mimickers (compounds that enhance FECD-associated expression patterns). These visualizations were generated by the authors using the L1000CDS2 ( https://maayanlab.cloud/L1000CDS2/ ) and SigCom LINCS ( https://maayanlab.cloud/sigcom-lincs/ ) web-based tools, which are freely available for academic research under open access principles. The visualizations shown are direct outputs from our analysis using these tools and are displayed under academic fair use principles and in accordance with the tools’ terms of use.

L1000CDS^2^ analysis identified 35 potential therapeutic compounds, ranked by their overlap scores (Fig. 3A). The highest-ranked compounds, including F1566-0341 (overlap score: 0.1074) and BRD-K92317137 (overlap score: 0.1063), demonstrated significant potential for reversing FECD-associated gene expression patterns. The expression signature heat map revealed that these compounds consistently reversed the direction of dysregulated genes by decreasing the expression of upregulated DEGs (blue) and increasing the expression of downregulated DEGs (red) (Fig. 3B).

SigCom LINCS analysis generated two distinct compound lists: 100 reversers and 100 mimickers (Fig. 3C, D). After removing duplicate entries, 76 unique reverser compounds were retained for further evaluation. These compounds were prioritized based on their potential to normalize FECD-associated gene expression patterns.

### Evaluation of cytoprotective effects of identified drug candidates

**Fig. 4:**
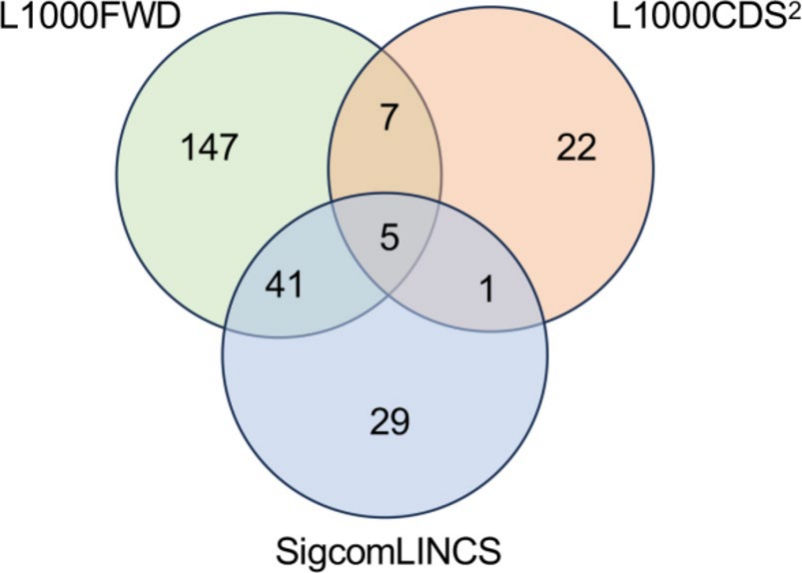
Identification of consensus drug candidates across three computational screening platforms. Venn diagram illustrating the overlap among drug candidates identified by three independent computational screening approaches: L1000FWD (200 drugs), L1000CDS^2^ (35 drugs), and SigCom LINCS (76 drugs). The analysis revealed five compounds common to all three platforms, representing the highest confidence therapeutic candidates for further evaluation. The diagram shows the complete distribution of drug candidates across platform intersections, highlighting the complementary nature of these screening approaches.

Comparative analysis using Venn diagrams revealed five compounds common to all three platforms (L1000FWD, L1000CDS^2^, and SigCom LINCS) (Fig. 4). These compounds were identified as cercosporin, LDN193189, menadione, BRD-K68313733, and BRD-A40431293 (Table 1). Among these, three commercially available compounds (LDN193189, cercosporin, and menadione) were selected for further evaluation.

**Fig. 5:**
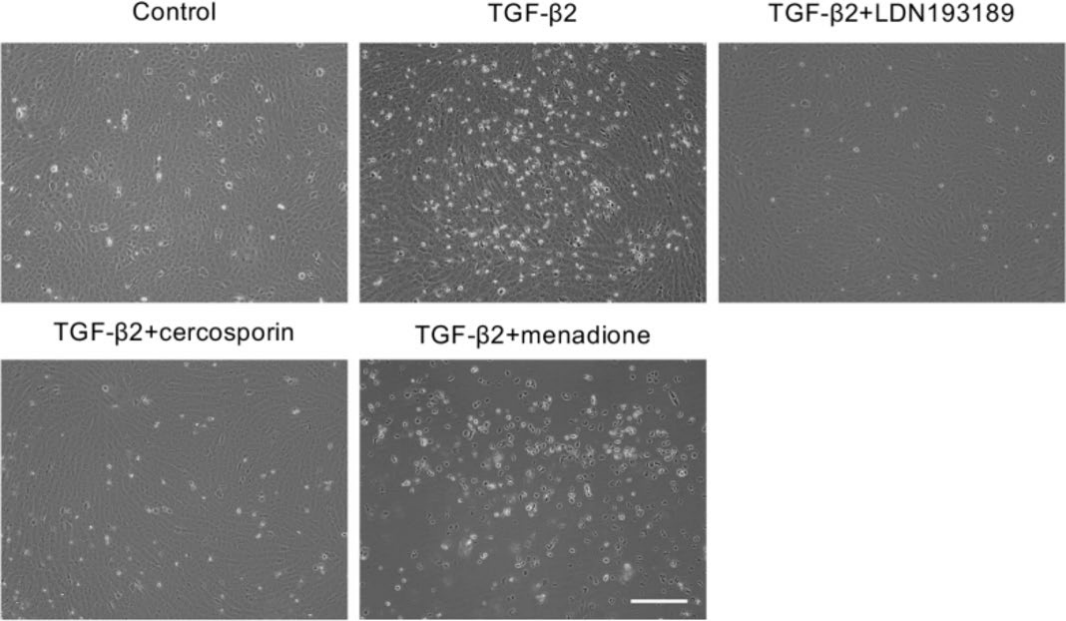
Morphological evaluation of drug candidate effects on TGF-β2–induced cell death in FECD model cells. Phase-contrast microscopy analysis of iFECD cells following drug treatment. Cells were pretreated with LDN193189 (1 µM), cercosporin (100 nM), or menadione (1 µM) for 24 h, followed by co-treatment with TGF-β2 (10 ng/mL) for 24 h. TGF-β2 treatment alone induced significant cell death, as evidenced by increased numbers of floating cells. Cotreatment with either LDN193189 or cercosporin markedly reduced TGF-β2-induced cell death, while menadione exacerbated cell death and induced widespread cell detachment, indicating cytotoxicity. Note that while LDN193189 and menadione were tested at 1 µM, cercosporin showed cytotoxicity at this concentration; therefore, a lower concentration of 100 nM was used based on preliminary studies demonstrating higher efficacy with reduced toxicity at this dose. Representative images from three independent experiments are shown. Scale bar: 100 μm.

The effects of these compounds were assessed in TGF-β2-stimulated iFECD cells using phase-contrast microscopy (Fig. 5). Cells were pre-treated with TGF-β2 for 24 h, followed by a 24 h exposure to one of the three test compounds. TGF-β2 treatment alone increased the number of floating dead cells compared to the control group. Co-treatment with TGF-β2 and either LDN193189 or cercosporin reduced TGF-β2-induced cell death. By contrast, menadione co-treatment exacerbated cell death and induced cell detachment, indicating cytotoxicity. Based on these observations, LDN193189 and cercosporin were selected for further investigation of their effects on excessive ECM production.

### Effects of LDN193189 on excessive ECM production

**Fig. 6:**
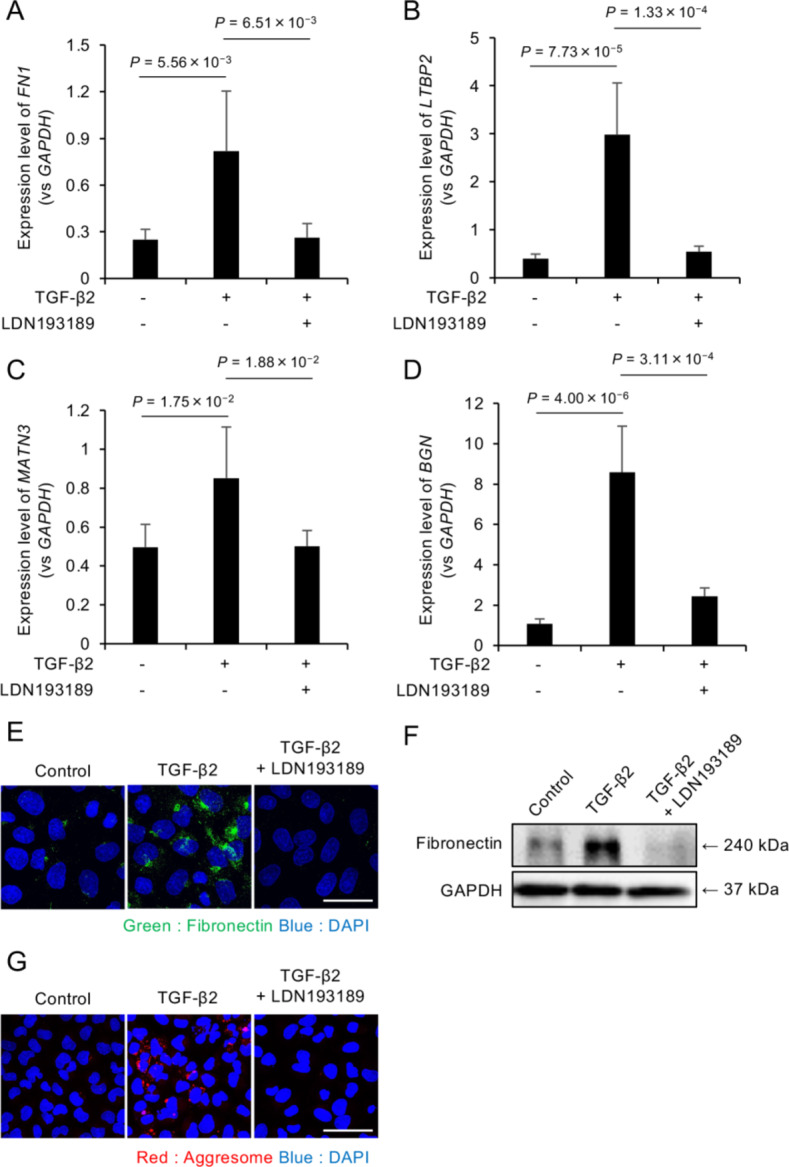
LDN193189 suppresses TGF-β2-induced ECM production and protein aggregation in FECD model cells. (**A-D**) Quantitative PCR analysis of ECM-related gene expression in iFECD cells pretreated with LDN193189 (1 µM, 24 h) followed by TGF-β2 stimulation (10 ng/mL). Expression levels of (**A**) FN1 , (**B**) LTBP2 , (**C**) MATN3 , and (**D**) BGN were significantly increased by TGF-β2 and suppressed by LDN193189 treatment. Data represent mean ± SD ( n = 5). Statistical significance was determined using Dunnett’s multiple comparison test ( P < 0.05). (**E**) Immunofluorescence analysis of fibronectin expression. Cells were treated as described above and stained for fibronectin (green) and nuclei (DAPI, blue). LDN193189 treatment normalized the TGF-β2–induced increase in fibronectin expression. Scale bar: 50 μm. (**F**) Western blot analysis confirming the suppressive effect of LDN193189 on TGF-β2–induced fibronectin protein expression. (**G**) Aggresome staining demonstrating the effect of LDN193189 on protein aggregation. Red fluorescence indicates protein aggregates, and nuclei are counterstained with DAPI (blue). Scale bar: 50 μm. Representative images from three independent experiments are shown in panels E-G.

The effects of LDN193189 on pathological ECM production were evaluated by pretreating iFECD cells with LDN193189 (1 µM) for 24 h prior to TGF-β2 stimulation (10 ng/mL). Quantitative PCR analysis revealed that TGF-β2 significantly increased the expression of the following ECM-related genes: *FN1* (Fig. 6A), *LTBP2* (Fig. 6B), *MATN3* (Fig. 6C), and *BGN* (Fig. 6D), whereas the LDN193189 treatment significantly suppressed the TGF-β2-induced upregulation of all four genes (*P* < 0.05 for all comparisons).

The effect on fibronectin protein expression was assessed using immunofluorescence staining and western blot analysis. Immunofluorescence revealed increased fibronectin expression following TGF-β2 stimulation, but this was normalized to control levels by LDN193189 treatment (Fig. 6E). Western blot analysis confirmed these findings, demonstrating that LDN193189 effectively suppressed TGF-β2-induced fibronectin upregulation (Fig. 6F). Given that pathological ECM proteins, such as fibronectin, can form unfolded proteins in FECD and lead to endoplasmic reticulum (ER) stress-induced cell death, aggresome staining was performed to assess protein aggregation. TGF-β2 treatment increased the accumulation of aggresomes, as indicated by enhanced red fluorescence. Cotreatment with LDN193189 markedly reduced this TGF-β2-induced protein aggregation (Fig. 6G), suggesting a protective effect against ER stress.

### Effects of cercosporin on excessive ECM production

**Fig. 7:**
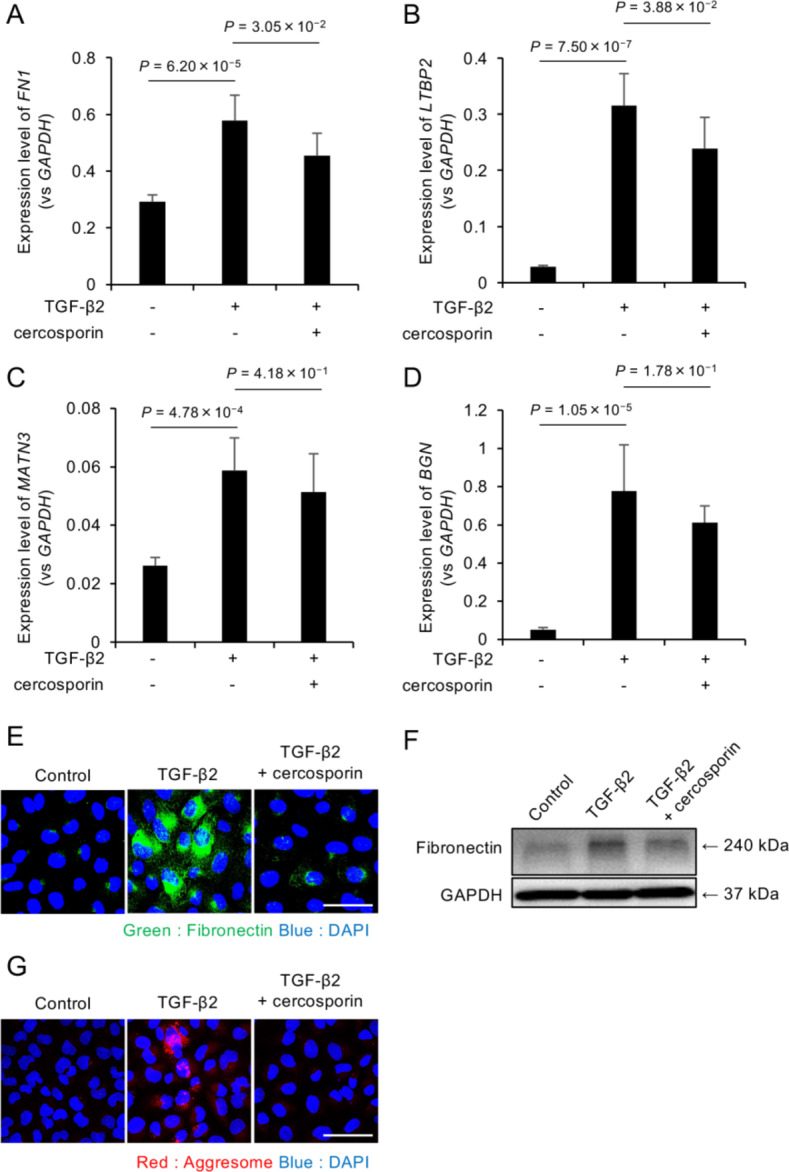
Cercosporin attenuates TGF-β2-induced ECM production and protein aggregation in FECD model cells (**A-D**) Quantitative PCR analysis of ECM-related gene expression in iFECD cells pretreated with cercosporin (100 nM, 24 h) followed by TGF-β2 stimulation (10 ng/mL). Cercosporin significantly suppressed TGF-β2-induced upregulation of (**A**) FN1 and (**B**) LTBP2 (P < 0.05). While (**C**) MATN 3 and (**D**) BGN expression showed downward trends in response to cercosporin treatment, these changes did not achieve statistical significance. Data represent mean ± SD (n = 5). Statistical significance was determined using Dunnett’s multiple comparison test. (**E**) Immunofluorescence analysis showing cercosporin-mediated suppression of TGF-β2–induced fibronectin expression (green). Nuclei were counterstained with DAPI (blue). Scale bar: 50 µm. (**F**) Western blot analysis confirming the inhibitory effect of cercosporin on TGF-β2–induced fibronectin protein expression. (**G**) Aggresome staining demonstrating the ability of cercosporin to reduce TGF-β2–induced protein aggregation (red). Nuclei are counterstained with DAPI (blue). Scale bar: 50 µm. Representative images from three independent experiments are shown in panels E-G.

Similar experimental conditions were used to evaluate the effects of cercosporin on ECM production. The cells were pretreated with cercosporin (100 nM) for 24 h before TGF-β2 (10 ng/ml) stimulation. Analysis of ECM-related gene expression showed that cercosporin significantly suppressed TGF-β2-induced upregulation of *FN1* (Fig. 7A) and *LTBP2* (Fig. 7B) (*P* < 0.05). Although *MATN3* (Fig. 7C) and *BGN* (Fig. 7D) expression also showed a downward trend in response to cercosporin treatment, these changes did not reach statistical significance. Immunofluorescence and western blot analyses of fibronectin expression demonstrated that cercosporin effectively normalized the TGF-β2-induced increase in fibronectin to control levels (Fig. 7E, F). Furthermore, aggresome staining revealed that cercosporin treatment prevented the TGF-β2-induced accumulation of protein aggregates, as indicated by the reduced red fluorescence intensity (Fig. 7G).

## Discussion

The identification of potential therapeutic targets for FECD is essential for developing pharmacological interventions that can prevent or slow the progression of this debilitating disease. In this study, we employed three computational drug screening platforms—L1000FWD, L1000CDS^2^, and SigCom LINCS—to identify compounds capable of normalizing the expression of DEGs associated with FECD. Through this approach, we identified LDN193189 and cercosporin as potential therapeutic candidates that could normalize FECD-associated DEGs. This combination of computational drug screening using disease-specific DEGs and cellular disease models represents a streamlined approach for identifying novel therapeutic compounds and can potentially accelerate the drug discovery process for FECD and other diseases.

These platforms are integral components of the NIH Library of Integrated Network-based Cellular Signatures program, and each offers unique capabilities for drug discovery^14^. From the various computational tools available for drug repurposing, we selected these three relatively common platforms because their complementary analytical approaches can enhance the precision of drug candidate selection. L1000FWD analyzes transcriptomic responses to over 20,000 compounds in human cell lines^11^and enables visual analysis of drug-induced gene expression responses and pattern recognition of mechanism of action (MOA), making it suitable for exploratory analysis. L1000CDS^2 ^processes over a million gene expression profiles using the characteristic direction method for improved signal-to-noise ratio^12^, allowing it to detect subtle gene expression changes with high accuracy and effectively identify drugs that can reverse disease-associated gene signatures. SigCom LINCS complements these platforms by integrating data from the LINCS, GTEx, and GEO databases^13^, providing cross-validation through multi-omics data integration and enhancing candidate reliability without dependence on specific cell lines.

In our research strategy, we employed L1000FWD for initial drug landscape analysis, L1000CDS^2 ^for screening drugs aimed at normalizing disease-related gene signatures, and SigCom LINCS for cross-validation through integrated multi-omics data. This approach maximized computational drug repositioning accuracy by leveraging each platform’s strengths. The utility of these platforms has been demonstrated across diverse therapeutic areas. They have been successfully employed in cancer research (including lung^15,16^, breast^17^, pancreatic^18^, and liver cancers^19^, chronic disease studies (such as Alzheimer’s disease^20,21^, rheumatoid arthritis^22^, and asthma^23^), and investigations of acute conditions (including COVID-19^24^and traumatic brain injury^25^). Despite this broad application and growing recognition in various medical fields, their utilization in ophthalmology has remained notably limited.

Our previous studies revealed distinct gene expression alterations in the FECD corneal endothelium and established their connection to disease pathology. These findings, combined with the proven success of these platforms in other therapeutic areas, motivated us to employ this computational approach to identify potential FECD therapeutic compounds. LDN193189, a small molecule inhibitor of bone morphogenetic protein (BMP) type I receptor kinases^26^, demonstrates diverse biological effects across multiple disease contexts. In cancer biology, it suppresses liver tumor-initiating cell characteristics by targeting CD133^27^and it inhibits colorectal cancer cell growth through suppression of autocrine BMP-4 signaling^28^. LDN193189 also shows promise in other therapeutic areas through its promotion of myoblast differentiation by inhibiting GDF8/myostatin signaling^29^and its attenuation of inflammation-associated anemia by reducing hepcidin expression^30^. Similarly, cercosporin, a photosensitizing toxin produced by cercospora fungi^31^, generates reactive oxygen species under light exposure^32^and has been studied for various therapeutic applications. A related compound, cercosporamide, has shown potential as an antifungal agent through its inhibition of Pkc1 kinase^33^.

While neither LDN193189 nor cercosporin has yet reached clinical application, both compounds show therapeutic potential across multiple applications. Our computational screening identified these compounds as normalizers of FECD-associated DEGs, although their precise mechanisms of action in this context remain to be elucidated. This represents a departure from conventional drug development approaches, which typically target specific molecular pathways with well-defined mechanisms of action. The challenge in determining specific mechanisms for these compounds may be inherent in our in silico screening approach, which prioritized global normalization of multiple DEGs rather than the modulation of individual pathways.

Based on the understanding that guttae formation in FECD results from excessive ECM production^1–3^, we investigated the effects of LDN193189 and cercosporin on pathological ECM expression. While guttae comprise multiple FECD-associated pathological ECM components, we focused on several key molecules: *FN1*, which has been well established in previous literature^34–36^, as well as *LTBP2*, *MATN3*, and *BGN*, which our previous shotgun proteomic analysis identified as upregulated genes in FECD patients^37^. Our findings demonstrated that both LDN193189 and cercosporin effectively suppressed the expression of these ECM proteins. Furthermore, these compounds showed inhibitory effects on unfolded protein accumulation. This observation is particularly significant given that excessive ECM production in FECD leads to unfolded protein formation^38,39^. Collectively, our results suggest that these normalizers may protect against cell death by reducing ER stress and subsequent dell death through the suppression of excessive ECM production.

However, this study has several limitations. Primarily, we have not fully elucidated the effects of these compounds on cell death mechanisms. FECD-associated cell death involves complex interactions between multiple pathways, including apoptosis^3,40,41^, ER stress^38,39,42^, and mitochondrial dysfunction^43^. Further investigation is needed to understand the precise mechanisms by which LDN193189 and cercosporin influence these cell death pathways. Additionally, the development of these compounds as FECD therapeutics requires preclinical studies using animal models^44^, which remains a crucial next step. Furthermore, as this was an exploratory study, we specifically targeted FECD patients with TCF4 TNR expansion > 50 repeats to focus on subjects with well-characterized genetic backgrounds. Whether the findings of this study can be applied to patients with fewer than 50 TNR expansions remains unknown at present.

The findings of this study highlight the potential of using computational drug screening as a powerful approach for identifying novel therapeutic candidates for FECD. While this approach has been well established in cancer research, our study demonstrates its applicability in ophthalmology. The combination of DEG-based computational drug screening and disease cell models provides an efficient pathway for therapeutic discovery that could be extended to other corneal endothelial diseases.

## Methods

### Ethics statement

This study adhered to the tenets of the Declaration of Helsinki and was conducted with the approval of the Ethics Committee of Friedrich-Alexander University Erlangen-Nürnberg (approval number: 140_20 B) and the Ethics Committee for Scientific Research Involving Human Subjects at Doshisha University (approval number: 20009). Written informed consent was obtained from all participants prior to tissue collection.

### RNA-Seq analysis of corneal endothelial cells

This study analyzed previously published RNA-Seq datasets and DEGs from corneal endothelial cells (CECs) of non-FECD and FECD subjects^9,45^. Analysis was restricted to FECD patients with trinucleotide repeat (TNR) expansion > 50 repeats, as confirmed by analysis of peripheral blood samples. Details of the control and FECD patient RNA-Seq datasets used in this study are as follows. Control samples were obtained from donor corneas of seven individuals (three Caucasian males and four Caucasian females, age range: 48–69 years)^45^. FECD patient samples were obtained from Descemet’s membranes containing corneal endothelial cells of six FECD patients with TCF4 TNR expansions who underwent corneal transplantation (three Caucasian males and three Caucasian females, age range: 53–79 years)^9^.

### In Silico **drug screening using L1000FWD**,** L1000CDS**^2^, **and SigCom LINCS**

Computational drug screening was performed using three platforms: L1000FWD (https://maayanlab.cloud/l1000fwd/)^11^, L1000CDS^2^ (https://maayanlab.cloud/L1000CDS2/#/index)^12^, and SigCom LINCS (https://maayanlab.cloud/sigcom-lincs/#/SignatureSearch/UpDown)^13^. Previously identified DEGs between FECD patients with *TCF4*TNR > 50 and non-FECD subjects were analyzed (706 upregulated and 962 downregulated genes)^9^.

The platforms identified two categories of compounds: reversers (compounds that normalize FECD gene expression patterns toward non-FECD levels) and mimickers (compounds that further amplify FECD-associated expression changes). For therapeutic potential, the analysis focused on reverser compounds (Fig. 1). The overlap of the identified reversers across all three platforms was visualized using Venn diagrams generated with the VennDiagram package (v1.7.3) in R (v4.0.3).

### Assessment of drug-mediated cytoprotection

A previously established immortalized corneal endothelial cell line derived from an FECD patient with confirmed *TCF4*TNR > 50 was used as the disease model (iFECD)^9,35^. This cell line was generated through immortalization of primary CECs using SV40 large T antigen and human telomerase reverse transcriptase (hTERT). The iFECD cells were maintained in Dulbecco’s modified Eagle’s medium (DMEM; Life Technologies Corp., Carlsbad, CA) supplemented with 10% fetal bovine serum and 1% penicillin-streptomycin (Life Technologies Corp.). Cells were passaged using 0.05% Trypsin-EDTA upon reaching 80% confluence.

For drug screening experiments, cells were grown to 80% confluence and serum starved in DMEM for 24 h. Following computational screening, selected compounds were evaluated for their effects on TGF-β2-induced pathological changes. The cells were treated with DMEM containing TGF-β2 (10 ng/ml; Wako, Osaka, Japan) for 24 h to induce excessive ECM production and endoplasmic reticulum-mediated cell death. The therapeutic potentials of LDN193189 (1 µM; Bio-Techne, Minneapolis, MN), cercosporin (100 nM; Adipogen Life Sciences, San Diego, CA), and menadione (1 µM; Adipogen Life Sciences) were assessed by adding them to the culture medium.

### Quantitative real-time PCR

Total RNA was isolated from cultured cells using the RNeasy Mini Kit (QIAGEN, Hilden, Germany). After washing the cells twice with Dulbecco’s phosphate-buffered saline (PBS) (Shimadzu, Kyoto, Japan), the cells were lysed in 350 µL Buffer RLT. The lysates were homogenized with an equal volume of 70% ethanol and purified using RNeasy Mini Spin Columns (10,000 × g for 15 s). Genomic DNA was removed by on-column digestion using RQ1 RNase-Free DNase (10 µL; Promega, Madison, WI, USA) in Buffer RDD (70 µL) for 20 min at room temperature. Following sequential column washing with Buffer RW1 (350 µL) and Buffer RPE (500 µL, twice), RNA was eluted in 50 µL RNase-free water and quantified using a NanoDrop spectrophotometer (Thermo Fisher Scientific Inc., Waltham, MA, USA).

For reverse transcription, 100 ng total RNA was used in a 20 µL reaction mixture comprising 5× RT Buffer (4 µL), 10 mM dNTPs (2 µL), 25 µM Random Primer (1 µL; Thermo Fisher Scientific Inc.), ReverTra Ace (1 µL; TOYOBO, Osaka, Japan), and RNase Inhibitor (1 µL; TOYOBO). Reverse transcription was performed at 30 °C for 10 min, followed by 42 °C for 1 min and 99 °C for 5 min.

The expression levels of target genes were quantified using TaqMan^®^ assays (Applied Biosystems, Foster City, CA) on a QuantStudio^®^ 3 Real-Time PCR System (Thermo Fisher Scientific Inc.). The TaqMan^®^ probes included *FN1* (Hs00365052_m1), *LTBP2* (Hs00166367_m1), *MATN3* (Hs00159081_m1), *BGN* (Hs00959141_g1), and *GAPDH* (Hs02786624_g1) as an internal control. PCR conditions consisted of initial denaturation at 95 °C for 20 s, followed by 40 cycles of 95 °C for 1 s and 60 °C for 20 s.

### Immunofluorescence and aggresome staining

The iFECD cells were seeded onto circular glass coverslips (Matsunami Glass, Osaka, Japan) in 24-well plates at a density of 8 × 10³ cells/well. Cells were fixed in 4% paraformaldehyde (PFA; Nacalai Tesque, Kyoto, Japan) for 10 min at room temperature and washed with PBS (Shimadzu). Cells were permeabilized with 0.5% Triton X-100 (Nacalai Tesque) and blocked with 2% bovine serum albumin (BSA; Nacalai Tesque) to prevent nonspecific binding. For immunofluorescence staining, specimens were incubated with anti-fibronectin antibody (1:15000; BD Biosciences, San Jose, CA), followed by Alexa Fluor^®^ 488-conjugated goat anti-mouse (1:1000; Life Technologies Corp.). Aggresomes were detected by incubating specimens with aggresome detection reagent (1:1000; Enzo Life Science Inc., Farmingdale, NY) for 45 min at room temperature. Nuclei were counterstained with 4’,6-diamidino-2-phenylindole (DAPI; Vector Laboratories, Burlingame, CA). Images were acquired using a DM 2500 fluorescence microscope (Leica Microsystems, Wetzlar, Germany).

### Immunoblotting analysis

The iFECD cells were lysed in ice-cold RIPA buffer (25 mM Tris-HCl [pH 7.6], 150 mM NaCl, 1% Nonidet P-40, 1% sodium deoxycholate, and 0.1% SDS) supplemented with Phosphatase Inhibitor Cocktail 2 (Sigma-Aldrich, St. Louis, MO, USA). Protein concentrations in the supernatants were determined using the bicinchoninic acid (BCA) protein assay (Thermo Fisher Scientific Inc.). Samples were denatured in 5× sample buffer at 95 °C for 5 min. Equal amounts of protein were separated by SDS-PAGE and transferred to FluoroTrans W PVDF Transfer Membranes (Pall Corporation, Port Washington, NY, USA). Membranes were blocked with 3% skim milk (Nacalai Tesque) in TBS-T (50 mM Tris [pH 7.5], 150 mM NaCl, 0.1% Tween 20) for 1 h at room temperature. Primary antibodies against fibronectin (1:15000; BD Biosciences) and GAPDH (1:3000; Medical & Biological Laboratories, Nagoya, Japan) were applied overnight at 4 °C. After incubation with horseradish peroxidase-conjugated anti-mouse secondary antibodies (1:5000; GE Healthcare, Chicago, IL, USA), protein bands were visualized using Chemi Lumi ONE Ultra (Nacalai Tesque) and analyzed using an Image Quant LAS 4000mini system (Fujifilm, Tokyo, Japan). Molecular weight markers (Bio-Rad, Hercules, CA) were included in all analyses.

### Statistical analysis

All statistical analyses were conducted using R software (version 4.3.0) with the multcomp library (version 1.4–25). Multiple group comparisons were performed using Dunnett’s multiple-comparisons test. Data are presented as mean ± standard deviation (SD), and statistical significance was defined as *P* < 0.05.

**Table 1:** Common drug candidates identified across three drug-Repurposing platforms.

Drug candidates	Description	L1000FWD (Similarity score)^†^	L1000CDS^2^ (Overlap)^#^	SigCom LINCS (z sum)^‡^
Cercosporin	Protein kinase C inhibitor	−0.0698	0.074	−19.27
LDN193189	Selective bone morphogenetic protein signaling inhibitor	−0.0999	0.101	−19.83
Menadione	Phosphatase inhibitor	−0.0762	0.083	−19.10
BRD-K68313733	N/A	−0.1257	0.086	−22.66
BRD-A40431293	N/A	−0.0816	0.078	−19.39

† Similarity score calculation: ratio of overlapping up/downregulated genes to effective input gene count. # Search score determination: proportion of shared differentially expressed genes (DEGs) between input and database, normalized by effective input size. ‡ Z-sum calculation: aggregate of z-scores from up- and downregulated gene sets, used for signature prioritization.

## Electronic supplementary material

Below is the link to the electronic supplementary material.

Supplementary Material 1

## Data Availability

The datasets used and/or analysed during the current study available from the corresponding author on reasonable request.
