# Supplementary material for: A feasibility of computational drug screening for Fuchs endothelial corneal dystrophy
Source: Sci Rep. 2025 Apr 26;15:14665. doi: 10.1038/s41598-025-95003-z (PMC12033358; doi:10.1038/s41598-025-95003-z)
Supplement: Supplementary file 1 — Supplementary Material 1 [file 41598_2025_95003_MOESM1_ESM.pdf]

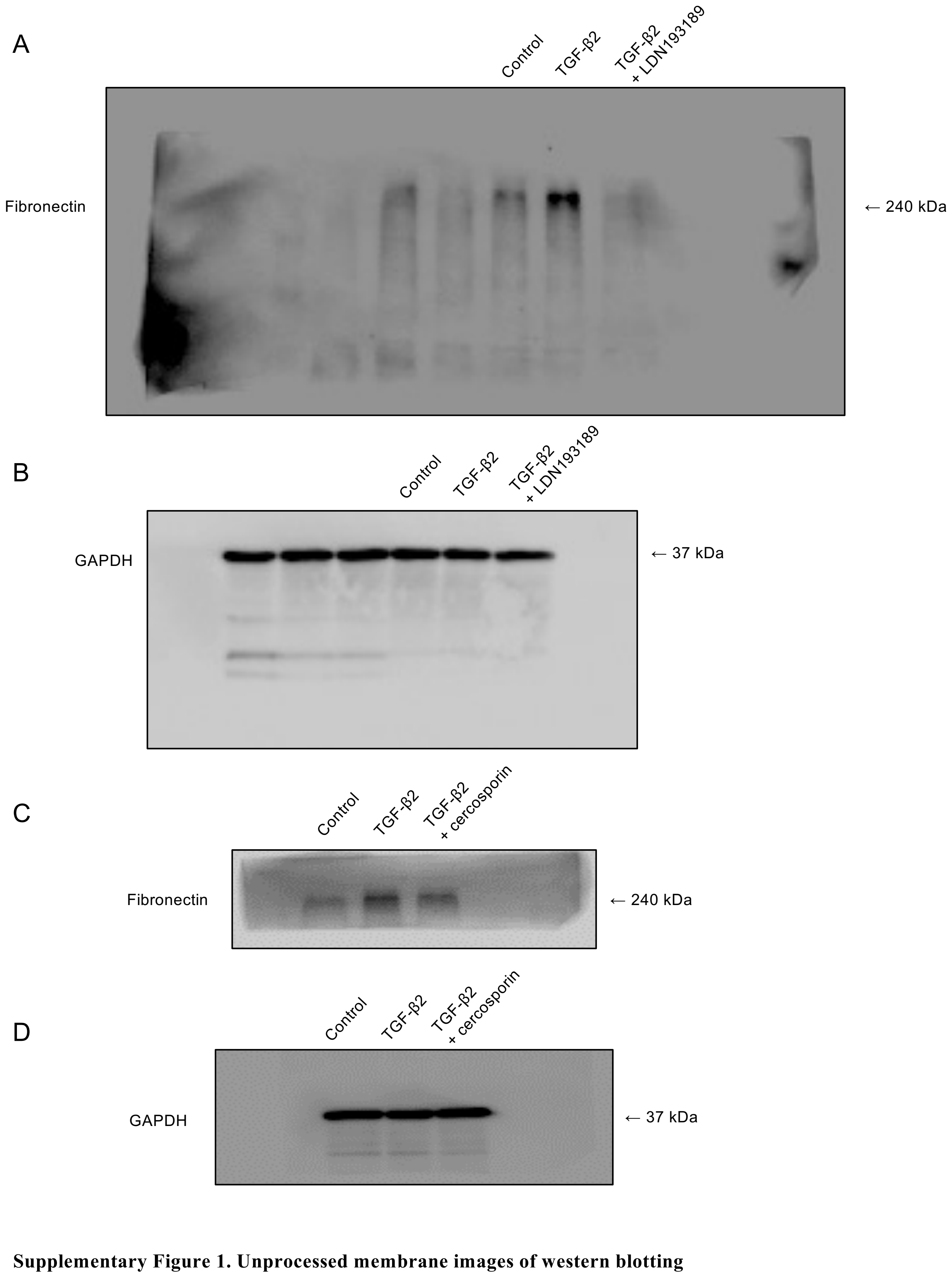

Supplementary Figure 1. Unprocessed membrane images of western blotting

A, B. Unprocessed full-length membrane images of western blotting analyses presented in Figure 6F. Panel A shows the original blot for Fibronectin, and panel B shows the original blot for GAPDH.

C, D. Unprocessed full-length membrane images of western blotting analyses presented in Figure 7F. Panel C shows the original blot for [target protein name], and panel D shows the original blot for GAPDH. All images are presented in their original, unprocessed versions to comply with transparency requirements. The positions of molecular weight markers are indicated on the left of each blot.
